# ‘A tough nut to crack’: inconsistent standards as roadblocks to data interoperability of health information systems in public hospitals in the Gauteng Province of South Africa

**DOI:** 10.1093/oodh/oqaf013

**Published:** 2025-07-01

**Authors:** Kabelo Given Chuma

**Affiliations:** Department of Information Science, College of Human Science, University of South Africa, Pretoria, South Africa

**Keywords:** standards, data interoperability, health information systems, data sharing, electronic health records, public hospitals

## Abstract

Standards are pivotal in achieving significant levels of data interoperability in the healthcare industry. However, inconsistent data standards and ambiguous guidelines stifle data interoperability in healthcare. Public sector hospitals in South Africa, particularly in Gauteng Province, face challenges in attaining data interoperability due to discrepancies in standards. This study investigates inconsistent standards as roadblocks to data interoperability of health information systems in public hospitals in the Gauteng Province of South Africa. A convergent parallel mixed methods research design was adopted, using an online questionnaire with 144 clinical and administrative personnel and semi-structured interviews with 16 managers. A multi-level sampling was used to select participants possessing the necessary expertise and experience in data interoperability and health information systems. Quantitative data were analyzed using SPSS for descriptive statistics, while qualitative data were thematically analyzed using the ATLAS.ti. The results indicated that hospitals in Gauteng adhere to multiple, conflicting standards, complicating data interoperability. Key factors contributing to this issue included legacy health systems, disparate systems, insufficient knowledge and awareness, weak regulations, and limited stakeholder collaboration. Furthermore, there was a notable lack of compliance with interoperability standards among hospitals. The study underscores the pressing need for coordinated efforts from policymakers, regulatory bodies, and health stakeholders to establish and enforce policies and standardized frameworks mandating uniform standards for interoperability. In conclusion, the cohesive implementation of uniform standards for data interoperability requires a holistic approach, incorporating clear policies, ongoing compliance monitoring, stakeholder collaboration, and continuous training to ensure the efficient exchange of healthcare data.

## INTRODUCTION AND BACKGROUND

The healthcare landscape is experiencing a significant transformation, fuelled by the urgent need for smooth data sharing and collaboration across health systems. Subsequently, data interoperability stands as a fundamental cornerstone of this evolution, promising seamless and meaningful exchange of patient data between different health systems and organisations to deliver patient-centric care at scale. [1, 2] advocate that data interoperability has become an essential aspect of healthcare to optimize patient care, boost efficiency, and streamline healthcare processes and delivery. This technology has the potential to revolutionize the industry by facilitating smooth data sharing, boosting patient engagement, and enhancing the overall quality of care [3]. As a consequence, standards are an essential prerequisite for data interoperability in healthcare. In particular, [4, 5] attest that the successful attainment of interoperability in the healthcare domain requires the creation, management, acceptance, and enforcement of realistic data and technical standards.

[6] emphasize that to achieve high levels of interoperability, hospitals, clinics, and other healthcare organizations must adhere to established standards and protocols such as European Committee for Standardization (CEN), General Data Protection Regulation (GDPR), International Organization for Standardization (ISO), Health Level Seven International (HL7), Integrating the Healthcare Enterprise (IHE), World Health Organization (WHO), Logical Observation Identifiers, Names, and Codes (LOINC), Systematized Nomenclature of Medicine – Clinical Terms (SNOMED), Clinical Data Interchange Standards Consortium (CDISC), World Wide Web Consortium (W3C) and Observational Medical Outcomes Partnership (OMOP). These standards are essential to provide a common language and a common set of expectations that enable data interoperability between health information systems and ensure compliance. In support of this assertion, [7] affirms that these data standards are designed to facilitate the development and use of data interoperability between healthcare information systems and care facilities. Despite the wide range of available standards, many healthcare organisations at various levels across the world encounter inconsistencies or incompatibilities in data standards across systems, which make it difficult to achieve data interoperability.

[8] stress that healthcare facilities in both high- and low-income countries fall short of achieving data interoperability due to the use of health information systems that adhere to different standards and protocols. For instance, a series of studies demonstrated that although some healthcare organisations have made significant efforts to achieve data interoperability; however, most of the healthcare facilities in developed countries such as Canada, the United States, Germany, Australia, and the Netherlands are still battling to achieve comprehensive health data interoperability because of the lack of comprehensive, centrally coordinated, fully validated, traceable and transmission standards [9–12]. On the other hand, many hospitals, clinics, and healthcare organisations in developing countries are facing similar situations and challenges in terms of attaining data interoperability. [13] affirm that many hospitals and primary healthcare clinics at the state level in Nigeria face significant challenges in achieving full interoperability, primarily due to inconsistencies in data standards. Similarly, [5, 14] caution that health centres in Uganda struggle with inconsistencies in standards, making it impossible to achieve data interoperability between systems. In contrast, [15] argue that Tanzanian public state hospitals are struggling to integrate data interoperability into their domestic health systems because of the absence of universal and harmonized standards.

These challenges in other countries are similar to the situation in South Africa, where many of the public sector hospitals and clinics use different systems with proprietary standards and protocols, making it difficult to share and interpret data across various platforms. While interoperability standards are available in South Africa and hospitals are expected to adhere to them, their adoption and implementation remain inconsistent. Many hospitals rely on different frameworks, further complicating efforts to achieve data interoperability [16]. In support of this assertion, the [17] attests that the lack of uniform data standards, inconsistent data formats, and the prevalence of legacy health systems further complicate interoperability efforts among South African public hospitals. Subsequently, [18, 19] argue that inconsistent or incompatible standards across South African healthcare systems lead to fragmentation, inefficiencies, and risks to patient safety. Based on this, addressing this issue is essential to prepare health information systems for swift, unified responses to future health emergencies.

While technical obstacles to data interoperability in South African healthcare have been well-documented in prior studies by [18, 20–22], there is, to the best of the researcher's knowledge, limited to no empirical study on how inconsistent standards directly impact the integration of data interoperability in public hospitals. Research in this area could provide valuable insights into how improved standardization might enhance the integration of data interoperability across healthcare systems. Therefore, the study seeks to address this gap by investigating the current landscape of data interoperability in public hospitals in Gauteng Province, examining the role of inconsistent standards, and identifying the challenges that hinder interoperability.

## PROBLEM STATEMENT

According to [23], data standards are considered the most critical building blocks for the interoperability of health information systems. At the most basic level, healthcare organisations must prioritize compliance with standards to successfully implement data interoperability between systems. Although standardization is seen as the key to achieving data interoperability, the unfortunate truth is that the South African healthcare sector is fraught with a plethora of challenges to integrate data interoperability between heterogeneous systems due to inconsistent data standards. The problem at hand may be articulated as the fact that public sector hospitals in the Gauteng Province of South Africa struggle to achieve data interoperability between EHR systems due to the vast number of data standards available, many of which are competing, overlapping, or even contradictory.

In support of this assertion, [24], along with [16, 22], underscore that many public hospitals and clinics in South Africa rely on diverse EHR systems that use incompatible data formats and conform to varying standards, posing significant challenges for achieving data interoperability, where data and information can be securely shared and accessed across platforms in hospitals. As a consequence, the absence of a certain level of consistent standards hinders the seamless sharing of health data, resulting in inefficiencies, delayed care, and compromised patient outcomes in public hospitals. [21] attest that the failure to establish and enforce consistent data standards in South African hospitals has led to fragmented health information systems, leading to data duplication, inconsistencies when accessing different EHR systems, and delayed service delivery. This fragmentation creates potential issues, including errors in diagnosis or treatment and delays to patient care while tests are repeated unnecessarily [25, 26].

Despite ongoing efforts by the Department of Health to modernize technical infrastructure and facilitate data exchange in South African healthcare, the absence of universally accepted, enforceable, and consistent standards continue to limit data interoperability within and across hospital systems. Therefore, this problem calls for urgent attention to standardize data protocols to achieve effective data interoperability in public hospitals. Although several studies have explored data standards for interoperability in South Africa, there is a gap in identifying scalable solutions for addressing inconsistent standards to achieving the data interoperability between health information in South African public hospitals. Thus, the study seeks to address this gap by investigating the current landscape of data interoperability in public hospitals in Gauteng Province, examining the role of inconsistent standards, and identifying the challenges that hinder interoperability

## RESEARCH OBJECTIVES

The following were research objectives of this study:

- To establish the existing standards for data interoperability adopted in public hospitals in Gauteng Province of South Africa
- To assess the level of compliance with existing standards for data interoperability in public hospitals in Gauteng Province of South Africa
- To identify the factors contributing to inconsistent standards for data interoperability in public hospitals in Gauteng Province of South Africa
- To analyze the consequences of inconsistent standards for data interoperability facing public hospitals in Gauteng Province of South Africa

## LITERATURE REVIEW

Data standards and related technology standards are essential to promote the development and use of data interoperability and facilitate the seamless sharing of information and data between health information systems. [7, 27, 28] assert that data and technical standards serve as essential tools and tangible outcomes for meeting the requirements necessary to enhance and achieve substantial levels of data interoperability among healthcare entities. These standards are crucial for ensuring that disparate systems, such as EHRs, can effectively share and understand data despite differences in technology or infrastructure.

According to [4, 29], there are several types of interoperability standards, each serving a distinct role in ensuring effective data exchange across systems. Nomenclature/terminology standards, such as SNOMED CT and ICD International Classification of Diseases (ICD), ensure that the same terms or codes are used to describe diagnoses, procedures, and other clinical data. Content and messaging standards, like HL7, FHIR, CDA, and DICOM, define how data is structured, formatted, and transmitted between systems [29]. These standards play a pivotal role in enabling systems to exchange data in a consistent and meaningful way. For example, HL7 is a widely adopted messaging standard used for the exchange of electronic health information, while FHIR (Fast Healthcare Interoperability Resources) facilitates the use of standardized, web-based data formats that allow easy access to healthcare data [30]. Other important interoperability standards include LOINC (Logical Observation Identifiers Names and Codes), which standardizes codes for laboratory observations, and CEN which develops standards for health data exchange in Europe [31]. OpenEHR is also among the most important standards in health informatics, providing a standardized, model-based approach to the representation and exchange of health information.

[32] underscore that OpenEHR facilitates interoperability through the use of open specifications, enabling healthcare organizations to adopt applications and systems from multiple vendors without being tied to proprietary platforms. It is important to note that OpenEHR is not a standalone EHR system itself, but rather a comprehensive, open standard specification that can be implemented to develop interoperable EHR systems [33]. This distinction is essential to avoid confusion, as OpenEHR defines the underlying architecture and models, while various systems built upon it, open source or commercial, serve as practical implementations of the standard. As a result, healthcare organizations can use open-standard systems from multiple vendors without becoming bound to one platform. These standards are essential in ensuring that healthcare organizations can share patient data securely and accurately, ultimately improving patient care and clinical decision-making. While standards like HL7, FHIR, and ICD are well-known, their adoption and implementation vary significantly across healthcare settings. [34] stipulate that in some countries, such as Estonia, Finland and Denmark, interoperability between EHR systems has been successfully achieved. In Estonia, for example, the country has implemented a nationwide e-health system where all health records are digital and integrated across both public and private healthcare providers. This success is attributed to the country's adoption of strict data standards, government-led initiatives, and a comprehensive regulatory framework that mandates the use of interoperable systems. Similarly, Denmark has established a robust infrastructure for EHRs, with a centralized health information exchange system that connects healthcare providers and ensures seamless data flow between them [35].

In contrast, many countries, including South Africa Nigeria, Zimbabwe, Uganda, and Tanzania face challenges in implementing these interoperability standards due to inconsistent adoption and varying compliance across healthcare organizations. According to [36], this inconsistency in applying standards results in fragmented data systems, leading to inefficiencies and risks to patient safety. [37, 38] argue that the lack of uniform adherence to interoperability standards significantly hinders effective communication and data exchange between systems, thus impeding the overall goal of seamless healthcare delivery. Similarly, [39] highlight that the lack of universally adopted healthcare interoperability standards is the biggest obstacle to achieving effective data exchange in the sector. [6] stress that failure to comply with established interoperability standards can result in significant challenges and failures in achieving seamless data exchange within healthcare facilities. The need for uniform standards is critical to reducing these challenges and improving healthcare outcomes.

Adherence to inconsistent interoperability standards poses significant challenges for healthcare organizations, with severe consequences spanning financial, legal, operational, and patient care domains. [40] emphasizes that such inconsistencies can lead to financial penalties, legal repercussions, reputational damage, business disruptions, diminished care quality, and erosion of trust. Similarly, [25] highlight the practical implications, including miscommunication among providers, redundant diagnostic tests, delayed treatments, and compromised patient safety. Complementing these findings, [36] underscore that inconsistent standards contribute to data interoperability failures, operational inefficiencies, and heightened risks to patient safety. Together, these insights reveal the critical need for uniform standards in healthcare.

In healthcare, maturity models and compliance frameworks are essential to understanding the progress and challenges of achieving data interoperability in healthcare which aligns to the objective of this study. According to [41], an interoperability maturity model can provide a structured approach to evaluating the progress and the challenges associated with achieving interoperability via an assessment of the progress made and the challenges to be overcome. [42] stress that interoperability maturity models, such as those from Interoperability maturity models, such as those from National Institute of Standards and Technology (NIST), categorize organizations into different levels, from basic interoperability with limited, manual data exchange to advanced interoperability, where data exchange is seamless and efficient. The models help healthcare organizations or countries determine what levels of interoperability they have attained and offer a roadmap for improving the level of interoperability in their organization or country.

## MATERIAL AND METHODS

### Study setting and design

This study was extracted from the Doctoral Thesis. The study was carried out at six public hospitals situated in the Gauteng Province of South Africa. This province was chosen because of its varied healthcare ecosystem, characterized by different degrees of technological advancement. The study adopted a convergent parallel mixed-methods design, integrating both quantitative and qualitative research approaches to comprehensively investigate inconsistent standards for data interoperability of health information systems in public hospitals in the Gauteng Province of South Africa. This approach was considered the most suitable for this study as it enabled a robust analysis by integrating statistical measurements with detailed insights from the study participants to capture a comprehensive understanding of the issues related to inconsistent standards for data interoperability in public hospitals.

### Participants

A multi-level sampling technique was employed to recruit participants, ensuring representation from various levels and categories within the study population. This approach allowed for the inclusion of diverse perspectives by selecting participants from different strata. In the quantitative phase, the study surveyed 144 administrative, clinical, and IT support staff working in various public hospitals across Gauteng Province. The sample size of 144 participants was determined based on recommendations from prior studies on similar healthcare-related research, where a similar number was found to be sufficient for achieving statistical power and ensuring generalizability of the findings within the context of the study. In the qualitative phase, a purposive sampling was used to select 16 administrative, IT, clinical, and records managers working in public hospitals. This sample size was chosen to ensure an in-depth understanding of the perspectives of key informants, as it allowed for a rich exploration of themes and provided diverse insights. The interviews were deemed adequate as they followed established guidelines for qualitative research, ensuring thematic saturation while balancing feasibility. The responses from participants in different hospital roles were analyzed by segmenting the data according to job function, allowing the study to capture variations in experiences and perceptions across different sectors of the healthcare system. The total sample size for this study was 160, as shown in Table 1.

**Table 1:** Breakdown of the target population

**Clinical and Administrative staff**	**No. of sample surveyed**	**Management staff**	**Codes**	**No. of sample interviewed**
Doctors	20	Clinical managers	CM	2
Nurses	17	Records managers	RM	3
Administrative clerks	23	IT managers	ITM	5
Network controllers	16	Administrative managers	AM	6
IT technicians	21			
Revenue clerks	12			
Registry clerks	16			
Ward clerks	8			
Data capturers	11			
**Total**	**144**	**Total**		**16**

(Source: Field Data 2022)

### Data collection

The quantitative data in this study were collected through an online questionnaire developed on Microsoft Forms. The questionnaire comprised Likert-scale items and closed-ended questions to enable statistical analysis. The online questionnaire was accessible for 90 days, from September 1 to November 30, 2022. Managers from each division were requested to collect and provide the email addresses and cell phone contact details of their colleagues. As a result, the necessary contact information was obtained, and a questionnaire link was distributed to key informants via email and WhatsApp. These key informants were encouraged to share the link with their colleagues, which contributed to achieving a higher response rate. On the contrary, qualitative data were collected through semi-structured interviews to gain deeper insights into challenges related to inconsistent standards for data interoperability. An interview guide was developed featuring open-ended questions designed to elicit detailed responses about experiences, perspectives, and specific challenges related to interoperability standards.

Policy documents, compliance reports, audit reports, and standard documents from the selected hospitals were reviewed to complement findings from the questionnaire and interviews. The interviews were conducted through face-to-face in participants’ offices and via Microsoft Teams. Each interview lasted between 20 to 30 minutes. All the interviews were conducted in English language. All interviews were audio and digitally recorded and transcribed verbatim to ensure data accuracy. Field notes were also recorded during the data collection process to offer additional context and enrich the analysis. Data saturation was reached by the 14th interview, with two additional interviews conducted to ensure sampling adequacy. In addition to questionnaires and interviews, direct observation of computer health systems and workflows was conducted during site visits to the participating hospitals. During the course of this investigation, computer systems, interfaces, and data exchange mechanisms were examined in various hospital units. The observation served as a validation step for cross-checking self-reported practices against real-world implementations and workflows. As a result of these insights, the overall triangulation strategy enhanced the credibility and contextual grounding of the study findings. Moreover, this allowed the researcher to validate participants’ responses and gain firsthand insight into system interfaces, data exchange, and workflow integration, highlighting key interoperability challenges.

### Data analysis

The collected quantitative data were analyzed using descriptive statistics and Statistical Package for the Social Sciences (SPSS) software (version 27) to generate counts, frequencies, and percentages. Thematic analysis was conducted using ATLAS.ti software (version 9.0) to systematically analyse the qualitative data. During the interpretation phase, the results of both the quantitative and qualitative data were integrated to offer a comprehensive understanding related inconsistent standards for data interoperability in hospitals. Insights from the analysis of the convergence and divergence between the two data sets were integrated with additional findings to offer a deeper and more comprehensive understanding of inconsistent standards impeding data interoperability in public hospitals. The use of a convergence parallel analysis enabled the researcher to examine the differences and seeking an explanation by comparing how both data sets contributed to understanding the phenomenon. The researcher considered the context and the different perspectives provided in the interviews to explain or resolve discrepancies in the quantitative findings.

### Validity, reliability, and rigor

To ensure the reliability of the instrument, the questionnaire was reviewed by clinical and IT managers, and adjustments were made based on their feedback, including paraphrasing certain items for clarity. A pilot study involving 30 study respondents was conducted to refine the questionnaire further. Furthermore, to enhance trustworthiness and minimise response bias in this study, a triangulation strategy was used by combining data from questionnaires, interviews, document reviews, and direct observation of hospitals systems. This enabled cross-verification and enhanced the credibility of the findings by linking self-reported data with actual practices. Trustworthiness in qualitative analysis was established through the independent exploration of narratives by two researchers, followed by a consensual validation of themes and sub-themes. To enhance the credibility of the study findings, member checking was conducted, allowing participants to confirm the accuracy of their responses. Additionally, qualitative data were converged with their quantitative counterparts for a comprehensive analysis, as recommended by [43].

### Methodological limitations

While the mixed-methods design and triangulation strategies enhanced the rigor of the study, several potential limitations should be acknowledged. First, the reliance on self-reported data in both the questionnaire and interviews may introduce response bias, despite efforts to validate findings through document reviews and direct observation. Second, digital literacy levels among some participants may have affected the accuracy or completeness of responses to the online questionnaire. Finally, recruitment challenges in busy hospital environments may have influenced participation rates, potentially limiting broader generalizability. These limitations were mitigated through pilot testing, follow-ups, and multi-source data triangulation.

### Ethical considerations

Ethical approval to conduct the study was granted by the College of Human Science Research Ethics Committee from the University of South Africa (Ref: 50119869_CREC_CHS_2022). Permission to collect data from public hospitals was approved by the Tshwane Research Ethics Committee (Ref: GP_202207_097). All procedures conducted in this study adhered to the ethical standards and principles outlined in the Helsinki Declaration, which were developed by the World Medical Association. Both written and verbal consent were obtained from all participants, ensuring their privacy and confidentiality were upheld throughout the study. Data security measures were implemented to safeguard sensitive information and comply with applicable regulations such as the Protection of Personal Information Act. Participants were informed of their right to withdraw from the study at any time, and no incentives were provided.

## RESULTS

This section presents the results for this study in accordance with the themes emerged from research objectives.

### Standards for data interoperability adopted in public hospitals in Gauteng Province, of South Africa

**Figure 1:**
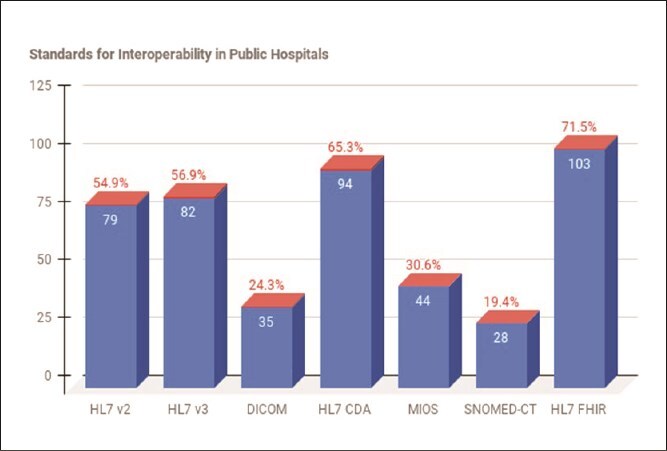
Standards for data interoperability in public hospitals (N=144)

According to [44], standards are fundamental requirements for data interoperability in healthcare. Respondents were asked to indicate the data standards adopted in public hospitals to support data interoperability. The results revealed that the majority of respondents 71.5% (103) reported that their hospitals adopted the HL7 FHIR standard. Additionally, a total of 65.3% (94) mentioned the HL7 CDA standard, 56.9% (82) the HL7 version 3 standard, and 54.9% (79) the HL7 version 2 standard. Other standards reported included the MIOM standard 30.6% (44), the DICOM standard 24.3% (35), and the SNOMED-CT standard 19.4% (28). These findings are illustrated in Figure 1.

To complement the data collected through the questionnaire, participants were interviewed to share their insights and experiences regarding the data standards that support and enable data interoperability in public hospitals. During the interviews, participants highlighted various data standards used in their hospitals to facilitate data interoperability between health information systems. For instance, the participant **(ITM-2)** said:

“Our hospital has adopted the HL7 CDA and SNOMED-CT as our main standards for structured messaging between health systems and application as well as for ensuring that data exchange between systems for seamless interoperability”

Participants **(RM-3)** and **(CM-1)** stated that:

“Our hospital adheres to several data standards to support the interoperability between systems including the MIOS, HL7 Version 2, and DICOM. We use these standards in most of our legacy computer systems for messaging and ensuring patient data is easily accessible when needed for clinical purposes”*.*

“Our EHR systems comply with prescribed norms and standards to support the seamless sharing of information and data across healthcare providers, such as HL Version 3, SNOMED-CT, and OpenEHR standards. We are heavily relying on these data standards to ensure that our care providers maintain the highest level of integrity, transparency and ethical conduct when delivering patient care”*.*

In contrast, some participants indicated that their hospitals had not adopted any standards to support data interoperability, while others were unaware of the data standards their hospitals had implemented. One interview participant **(AM-1)** stated:

“To be honest with you, our public hospital has not yet implemented data standards to support or promote the data interoperability between health information systems”

Participant **[CM-2]** said:

“I have no idea what data standards our hospital adheres to for data interoperability. However, we have hospital documents and manuals that outline the most important information relating to interoperability standards, which you might find helpful if you review and refer to them”*.*

Furthermore, the document analysis revealed that public hospitals adhere to a variety of standards that promote data interoperability, including HL7 v2, HL7 v3, HL7 CDA, DICOM, OpenEHR, SNOMED-CT, and HL7 FHIR.

### Level of compliance with existing standards for data interoperability in public hospitals in Gauteng Province of South Africa

**Figure 2:**
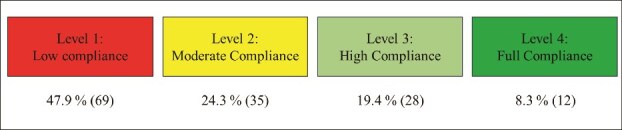
Level of compliance with standards for data interoperability in public hospitals (N=144)

According to [45], ensuring strict adherence to standards is crucial for healthcare organizations to maintain quality, safeguard patient safety, operate ethically, and uphold public trust. On a scale of 1 to 5, respondents were asked to indicate the extent to which they believe their hospitals comply with existing standards to facilitate data interoperability between health information systems. The majority of respondents 47.9% (69) rated the compliance level as 1, showing no compliance with existing data standards for interoperability. 24.3% (35) rated the compliance level at 2, indicating moderate compliance. While only 19.4% (28) indicated a compliance of 3, showing high compliance but not complete adherence. A small portion of the respondents 8.3% (12) rated the compliance level as 5, indicating that their hospitals have full compliance with the existing standards for interoperability as shown in Figure 2

In the interviews, most of the participants expressed inadequate compliance among public hospitals and staff with existing standards designed to support data interoperability between health information systems. For example, participants **(ITM-4), (RM-1), (AM-5), and (AM-2)** stated that:

“I have realised that our hospital has adopted several data standards such as the HL7 CDA and DICOM, but the compliance is patchy. Most of our departments and other hospital units do not adhere to the guidelines consistently, which causes data-sharing issues between our systems and interoperability failure.''

``Our compliance with technical standards like OpenEHR and SNOMED-CT is so poor that even simple tasks like transferring patient records between hospitals in the Gauteng Province become a challenge, leading to delays in patient care.''

"Compliance with HL7 standards is poor because the IT systems in use are not regularly updated, which means data exchange fails frequently. And again, the reason behind this poor compliance is due to insufficient training provided to staff and some departments in the hospital ignore them, making interoperability impossible to achieve”.

“The hospital's compliance is minimal because most of our staff have little awareness about the technical standards such as DICOM, SNOMED-CT, HL7 CDA and this lack of knowledge results in serious challenges to achieve data interoperability within hospitals in the Gauteng Province”

On the contrary, two participants **(ITM-4)** and **(ITM-5)** said that the level of compliance with standards that promote data interoperability is good and satisfactory.

“In our hospital, compliance with standards that support data interoperability has been a priority for us, and we have allocated resources to ensure proper implementation and monitoring. In addition to this, our staff receive regular training to ensure that they comply with existing standards, legislation, and policies”

``Our hospital has established a compliance audit team and integrated mechanism that ensures all health information systems are aligned with data standards and protocols. This has significantly boosted our interoperability efforts''

### Factors contributing to inconsistent standards for data interoperability in public hospitals in Gauteng Province of South Africa

**Figure 3:**
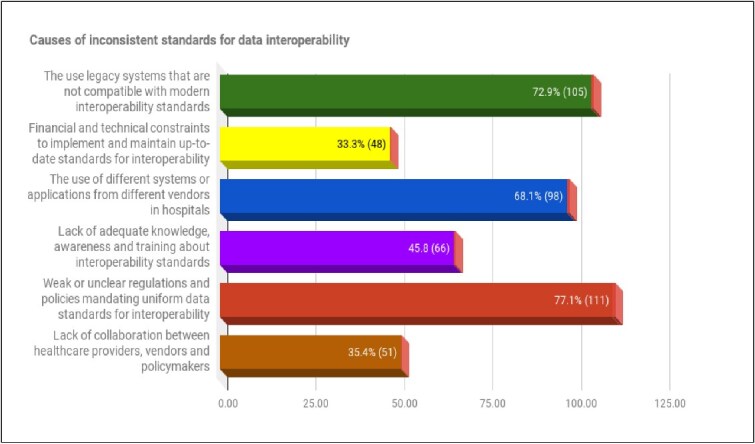
Causes of inconsistent standards for data interoperability in public hospitals (N=144)

There are numerous factors that contribute to the inconsistency of data standards that hinder the successful adoption and implementation of data interoperability in healthcare settings. In the questionnaire, respondents were asked to identify the causes or factors they believe contribute to the issue of inconsistent standards for interoperability within their respective hospitals. Figure 3 illustrates that the majority of respondents 77.1% (111), pointed weak or unclear regulations and policies mandating data standards as a primary cause. Moreover, 72.9% (105) highlighted the use of legacy systems and outdated software in hospitals as a contributing factor. A significant proportion, 68.1% (98) attributed the issue to the use of different disparate systems or applications from different vendors, leading to inconsistent data standards for interoperability. A total of 45.8% (66) of respondents cited a lack of adequate knowledge and awareness among staff, as well as insufficient regular training on interoperability standards. Financial and technical constraints in implementing and maintaining up-to-date data interoperability standards were identified by 35.4% (51). Furthermore, the minority of respondents 33.3% (48) cited the lack of collaboration between healthcare providers, vendors, and policymakers as a contributing factor to the inconsistency of data standards across hospitals.

To complement the data collected through the questionnaire, participants were interviewed to share their insights on the factors they believe contribute to the use of inconsistent standards for data interoperability in their public hospitals. Most of the participants indicated several causes of inconsistent standards for data interoperability in hospitals. For instance, the participant **(ITM-1)** said:

“Personally, I think our South African government is failing us because we do not have policies, procedures, and clear guidelines in place to emphasise and ensure that all public hospitals across Gauteng Province use the same standards for supporting data interoperability. So, we need clear and approved regulations and policies that will encourage the use of universal standards across all hospitals”*.*

Another participant **(ITM-2)** and **(AM-6)** indicated that:

The most common issues that contribute to inconsistent data standards is because our hospitals use different systems that conform to different formats and protocols. Therefore, this forces our hospitals to use different standards and rules for interoperability and results in inconsistent standards among hospitals and causes a lot of fragmentation.

“The problem is that our healthcare stakeholders nationwide fail to work together effectively to establish a uniform approach to ensure strict compliance with consistent standards for data interoperability. So…in a nutshell, I believe that the lack of coordination is the primary reason our hospitals in this province struggle to successfully achieve universal standards for data interoperability”

### Consequences of inconsistent standards for data interoperability among public hospital in Gauteng Province of South Africa

**Figure 4:**
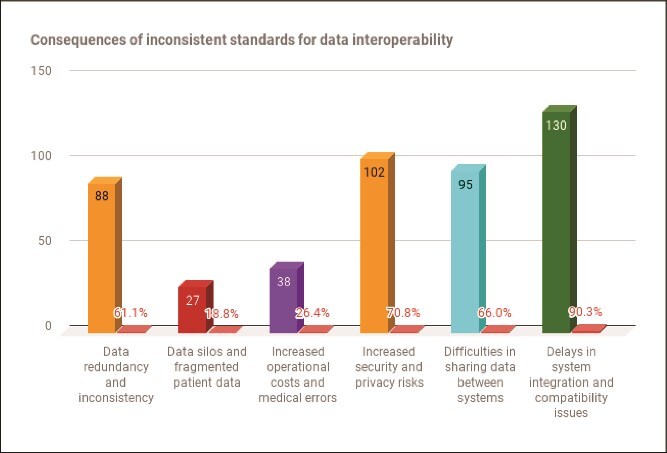
Consequences of inconsistent standards for interoperability in public hospitals (N=144)

According to [46], inconsistent standards and protocols can result in significant challenges, hindering healthcare organizations from achieving genuine and effective interoperability between systems. In the last section of the survey questionnaire, respondents were asked to identify the consequences their public hospitals had experienced due to inconsistent standards for data interoperability. The results in Figure 4 illustrate that an overwhelming majority of respondents 90.3% (130) reported delays in system integration and compatibility issues. The majority of respondents 70.8% (102) highlighted increased security and privacy risks, while 66.0% (95) cited challenges in sharing information and data between systems. Furthermore, a total of 61.1% (88) mentioned data redundancy and inconsistencies, 26.4% (38) reported increased operational costs and medical errors, and a smaller proportion of respondents 18.8% (27) identified data silos and fragmented patient data as consequences.

To supplement the data gathered through the questionnaire, participants were interviewed and asked to share their experiences regarding the consequences their public hospitals faced due to inconsistent standards in promoting data interoperability between health systems. Participants mentioned several consequences their hospital experienced, including unnecessarily increased costs, redundant clinical and administrative processes, fragmented health systems, security and privacy issues, and compatibility issues. For example, the participant (RM-1) said:

Our hospital is experiencing serious difficulties in integrating new health systems and compatibility issues because most of our existing systems are outdated and don’t adhere to common standards. These delays make us stacked with outdated technology and infrastructure. The government and the Department of Health must introduce universal standards and ensure that all hospitals around Gauteng adhere to the same standards to be able to achieve interoperability and integration.

In contrast, other participants (AM-1), (AM-2), (ITM-3) mentioned that:

During my time working here, I have observed that our hospital faces numerous critical challenges, including significant delays in service delivery, unnecessary cost increases, and redundant clinical and administrative processes. These issues stem from the lack of uniform and consistent standards necessary to achieve full interoperability between our health information systems.

The systems in our hospitals adhere to different data standards, which hinders their ability to communicate and share information effectively. This fragmentation forces our facilities to operate in silos, resulting in inefficiencies and posing significant risks to patient outcomes.

Our systems do not consistently align with the latest standards, making it challenging for our hospital to achieve interoperability between EHR systems. This misalignment increases the risk of failing to meet privacy and security requirements, particularly concerning patient data.

## DISCUSSION

This section discusses the findings collected through survey questionnaire and interviews. The findings from the quantitative phase reveal that hospitals in the Gauteng province adhere to various standards that enable data interoperability between health information systems, including HL7 v2, HL7 v3, HL7 CDA, and HL7 FHIR. The qualitative findings add depth to these results, revealing that hospitals adopted the HL7 CDA and HL7 v2. Furthermore, the qualitative findings highlight the adoption of other standards not identified in the quantitative phase, such as SNOMED-CT, MIOS, DICOM, and OpenEHR. This is consistent with a previous study by [47] who established that HL7, CDA, HIPAA, SNOMED-CT, DICOM, CEN, and OpenEHR as the most critical data standards for developing and implementing interoperability between health information systems in hospitals. These findings align with the study conducted by [48], which found that health facilities in Uganda adhere to interoperability data standards such as HL7, FHIR, and DICOM. However, they contrast with findings from [49], which revealed that healthcare facilities in Australia follow interoperability standards such as OMOP-CDM, LOINC, CDISC, and HL7 FHIR. This divergence confirms that different hospitals across the globe adhere to different standards designed to support interoperability. The findings from the questionnaire, in conjunction with insights gained from the interviews, revealed a significant lack of compliance among hospitals with standards intended to facilitate data interoperability.

This finding aligns with the findings of a study by [48, 50] who established that that healthcare facilities in Uganda face numerous challenges in achieving data interoperability and integration. These challenges stem from system fragmentation, the lack of universal standards, and the failure to adhere to standardization requirements essential for implementing data interoperability across healthcare settings. In contrast, [51] stress that healthcare systems in the United States, United Kingdom, and Canada demonstrate strong compliance with standards that facilitate data interoperability among hospitals and healthcare organizations. Based on the findings, it is clear that the healthcare system in South Africa is lagging behind in terms of ensuring compliance with standards, which is the primary reason for the failure of data interoperability. The quantitative findings demonstrate several key factors contributing to inconsistent standards for data interoperability among public hospitals. These include reliance on legacy systems incompatible with modern standards, the adoption of disparate systems from various vendors, and unclear regulations and policies mandating uniform data standards. Similarly, qualitative insights reinforce these findings, revealing that the absence of clear policies and guidelines, the use of diverse systems adhering to different formats and protocols, and limited collaboration among healthcare stakeholders exacerbate the inconsistency in data interoperability standards across hospitals.

The findings are in agreement with [52–55] who emphasised that the absence of regulatory frameworks to enforce compliance, inconsistent technical specifications across systems, significant gaps in national policies, and a lack of comprehensive guidance, limited coordination and collaboration between stakeholders further exacerbate the problem of inconsistent interoperability standards in healthcare. Moreover, the findings from the quantitative phase revealed that public hospitals had experienced several adverse consequences due to inconsistent data standards for interoperability. These include data redundancy and inconsistency, heightened security and privacy risks, delays in system integration, and compatibility issues. Complementing these insights, the qualitative findings highlighted additional consequences faced by public hospitals, such as service delivery delays, unnecessary cost escalations, redundant clinical and administrative processes, system fragmentation and silos, as well as increased security and privacy vulnerabilities.''

## RECOMMENDATIONS

This study makes the following recommendations based on the findings:

- Policymakers and regulatory bodies must develop and enforce formal written policies and regulatory frameworks mandating uniform standards for data interoperability of health information systems across public hospitals. These policies and frameworks should specify technical and procedural requirements for adopting data interoperability standards.
- Healthcare stakeholders and policymakers should implement a unified approach to data standards and protocols by adopting universally acceptable data standards such as HL7 FHIR and DICOM, and ensure public hospitals transition away from disparate systems and legacy platforms.
- Public hospitals must prioritize mandatory training and development programs to educate health staff and IT personnel to enhance their understanding of and compliance with data interoperability standards, policies, and regulations.
- The Department of Health together with the government should establish a centralized monitoring and evaluation framework to oversee the adoption and implementation of data interoperability standards in hospitals. This mechanism should conduct regular compliance reviews, address identified challenges, and ensure alignment with established guidelines.
- Public hospitals should mandate IT and software vendors to comply with defined interoperability standards when developing and deploying health information systems to ensure seamless integration across platforms.
- The South African government must provide financial and technical resources to public hospitals to support the transition to interoperable systems, including investment in modern IT infrastructure and support for system integration initiatives.

## CONCLUSION

This study sought to investigate the inconsistent standards as roadblocks to data interoperability of health information systems in public hospitals in the Gauteng Province of South Africa. The findings revealed that public hospitals adhere to various data standards, including HL7 v2, HL7 v3, HL7 CDA, DICOM, OpenEHR, SNOMED-CT, and HL7 FHIR. Based on this finding, it can be concluded that coexistence of multiple standards within hospitals create complexities, leading to inconsistencies in data exchange and integration processes. The findings from both the questionnaire and interviews revealed a critical issue of poor compliance among public hospitals with the standards necessary for ensuring data interoperability. This lack of adherence to established standards contributes to inefficiencies and challenges in achieving seamless data exchange across healthcare systems. The findings further indicated key factors contributing to inconsistent data interoperability standards among public hospitals, reliance on outdated legacy systems that are incompatible with modern standards, the use of varied systems from different vendors, limited collaboration among healthcare stakeholders and the lack of clear regulations and policies requiring uniform data standards. It is worth noting that these factors collectively contribute to the fragmentation and inconsistency in data interoperability standards across public hospitals.

The findings revealed several key challenges faced by public hospitals due to inconsistent data interoperability standards, including data redundancy, security and privacy risks, delays in system integration, and compatibility issues. Additional challenges include service delivery delays, increased costs, redundant processes, system fragmentation, and greater vulnerabilities. These findings underscore the significant operational and security challenges that arise from inadequate data interoperability standards in healthcare settings. This study makes a significant contribution to the field of health systems by providing an in-depth analysis of the challenges posed by inconsistent standards for data interoperability in public hospitals. The findings highlight how the lack of uniformity in data standards hampers the ability to achieve seamless interoperability within South African public hospitals. By focusing on these inconsistencies, the study enhances our understanding of the barriers to effective data sharing and integration in healthcare settings. Ultimately, this study contributes to the literature while offering practical recommendations to address interoperability issues, improving the operational efficiency and security of healthcare systems.

This study had its limitations like any research. The main limitation is that the study was confined to public hospitals in the Gauteng province, excluding private hospitals and public hospitals from other provinces. This limitation may affect the generalizability of the results to the broader healthcare system in South Africa. Therefore, future studies should consider expanding the scope to include private and public hospitals from other provinces across South Africa. This broader approach could provide a more holistic understanding of the challenges associated with data standards for data interoperability across diverse healthcare settings, thereby enhancing the applicability and relevance of the findings to the national context. In conclusion, the study underscores the pressing need for policymakers, regulatory bodies, and the government to enforce policies and standardized frameworks mandating uniform data interoperability standards for health information systems across public hospitals. By adopting and enforcing universal standards, hospitals can strive to achieve data interoperability, enhance operational efficiency, and improve patient healthcare outcomes.

## Data Availability

The data underlying this article will be shared on reasonable request to the corresponding author.
